# Potential New Target for Dry Eye Disease—Oxidative Stress

**DOI:** 10.3390/antiox13040422

**Published:** 2024-03-29

**Authors:** Jinghua Bu, Yanbo Liu, Rongrong Zhang, Sijie Lin, Jingbin Zhuang, Le Sun, Lingyu Zhang, Hui He, Rongrong Zong, Yang Wu, Wei Li

**Affiliations:** 1 Department of Ophthalmology, Xiang’an Hospital of Xiamen University, Eye Institute of Xiamen University, Fujian Provincial Key Laboratory of Ophthalmology and Visual Science, Fujian Engineering and Research Center of Eye Regenerative Medicine, School of Medicine, Xiamen University, Xiamen 361005, China; 2 Zhongshan Hospital (Xiamen), Fudan University, Xiamen 361015, China; 3 Xiamen University Affiliated Xiamen Eye Center, Xiamen 361102, China

**Keywords:** dry eye disease, oxidative stress, reactive oxygen species, oxidants, antioxidants

## Abstract

Dry eye disease (DED) is a multifactorial condition affecting the ocular surface. It is characterized by loss of tear film homeostasis and accompanied by ocular symptoms that may potentially result in damage to the ocular surface and even vision loss. Unmodifiable risk factors for DED mainly include aging, hormonal changes, and lifestyle issues such as reduced sleep duration, increased screen exposure, smoking, and ethanol consumption. As its prevalence continues to rise, DED has garnered considerable attention, prompting the exploration of potential new therapeutic targets. Recent studies have found that when the production of ROS exceeds the capacity of the antioxidant defense system on the ocular surface, oxidative stress ensues, leading to cellular apoptosis and further oxidative damage. These events can exacerbate inflammation and cellular stress responses, further increasing ROS levels and promoting a vicious cycle of oxidative stress in DED. Therefore, given the central role of reactive oxygen species in the vicious cycle of inflammation in DED, strategies involving antioxidants have emerged as a novel approach for its treatment. This review aims to enhance our understanding of the intricate relationship between oxidative stress and DED, thereby providing directions to explore innovative therapeutic approaches for this complex ocular disorder.

## 1. Introduction

Dry eye disease (DED), a multifactorial and complex disease of the tear film, eyelids, lacrimal glands, and a variety of ocular surface tissues, is characterized by loss of tear film homeostasis and accompanied by ocular symptoms [1]. Its multifaceted etiology includes factors such as systemic inflammatory diseases, localized eye problems, or commonly used medications [2]. DED is classified into two primary categories of aqueous deficient and evaporative, both contributing to hyperosmolality and tear film instability [3,4]. Estimates suggesting that 5% to 50% of the global population suffers from DED have drawn significant attention to this disease [5,6]. The clinical presentation of DED can vary widely, but common symptoms include persistent grittiness, ocular redness, burning, itchiness, foreign body sensation, eye fatigue, and visual disturbance [1,3]. As a chronic condition, DED can substantially impact on quality of life, as it affects not only visual function but also comfort and overall wellbeing [7,8,9].

In clinical practice, the diagnosis and treatment of DED can be challenging due to its complex pathophysiology. Despite the existence of a variety of treatment strategies, DED often persists due to incomplete treatment. Ocular lubricants, applied in an attempt to replace and/or supplement natural tear film, are considered a mainstay of DED therapy. However, they only provide temporary relief from the symptoms of DED and do not address its underlying causes [10]. Furthermore, inflammation has been identified as a pivotal factor contributing to the vicious cycle of DED; thus, effective control of inflammation constitutes a crucial step in preventing and treating chronic DED [11,12]. Corticosteroids, as the most commonly used anti-inflammatory drugs, may give rise to several complications when employed over an extended duration, including glaucoma and cataract [10]. The immunomodulatory agents used in approved therapies for DED, such as cyclosporine and lifitegrast, effectively inhibit T-cell activation and cytokine production, but their effectiveness varies among DED patients [13]. Therefore, the limited efficacy of the current treatment approaches underscores the pressing need for innovative therapeutic strategies that address the multifaceted nature of this condition [14].

Inflammation represents a breakthrough point in the vicious cycle of DED. However, it has recently been found that relying solely on inflammation inhibition for the treatment of multifactorial and chronic diseases such as DED might compromise therapeutic efficacy. This is because there exists an upstream mediator in the form of excessive reactive oxygen species (ROS) production, which is characterized as oxidative stress [15,16]. The concept of oxidative stress has garnered increasing attention within the field of ophthalmology, emerging as a novel avenue for the treatment of DED. Oxidative stress is a process wherein the antioxidant defenses of cells become overwhelmed by the generation of ROSs [17]. At physiological concentrations, ROSs induce the appropriate level of oxidative stress required for a range of cellular processes, such as apoptosis, inflammation, innate immunity, and wound healing, as well as the regulation of transcription factors and their surface receptors [18]. However, excess intracellular ROS production can lead to the oxidation of DNA, proteins, lipids, and metabolites, thereby leading to possible disruptions in gene and protein expression patterns, protein aggregation, and cellular dysfunction [19]. The eye is extensively exposed to light and robust metabolic activity, and it encounters high oxygen tension in specific regions, rendering it particularly susceptible to oxidation imbalance [20,21]. In clinical studies, proteomic analysis of tears from DED patients has revealed increased oxidative activity, accompanied by the upregulation of proteins associated with oxidative stress injury [22]. ROS production has been detected in various models of in vitro studies [23,24]. RNA-seq analysis of corneal epithelial cells exposed to hyperosmolarity has revealed significant modulation in the activation of ROS metabolism processes [25]. In humans, numerous exogenous and endogenous factors are sources of ROS production, which was particularly emphasized in a recent cross-sectional study on population aging and lifestyle factors, where it was found that DED is associated with aging, reduced sleep duration, prolonged exposure to digital device screens, and increased psychological stress [26]. Aging primarily arises from internal metabolic processes, often the relatively reduced presence of antioxidant defenses on the ocular surface [27], an inevitable factor that has been extensively studied for a considerable duration. Moreover, an elevation in oxidative stress markers was observed in the lacrimal and meibomian glands of elderly mice [28]. The risk of developing DED not only increases gradually during the aging process, but also warrants attention due to the changing lifestyle of modern young individuals. For example, the common and essential practice of digital displays in the daily lives of young people has been shown to alter oxidative stress markers in the tear film, inflammatory mediators, and tear osmolarity of computer users [29,30]. Thus, the risk of developing DED persists across different age groups. Furthermore, fluctuations in hormonal levels and frequent smoking and alcohol consumption can also increase oxidative stress levels on the ocular surface [31,32]. Collectively, these factors emphasize the substantial role of oxidative stress in the development of DED.

For this review, we searched the PubMed database using the terms ‘oxidative stress’, ‘ROS’, ‘dry eye’ or ‘dry eye disease’, and ‘antioxidants’ to find articles published between 2010 and 2024. We included in vitro, in vivo, experimental, and clinical studies and excluded leader opinion, descriptive studies, and case series reports. In this review, we comprehensively explore the intricate relationship between oxidative stress and DED, spanning from basic research to potential clinical applications. As such, we aspire to deepen the understanding of the pathogenic mechanisms involved in DED, paving the way for the development of innovative therapeutic approaches and strategies to better address the clinical needs of patients.

## 2. Relationship between Oxidative Stress and DED

**Figure 1:**
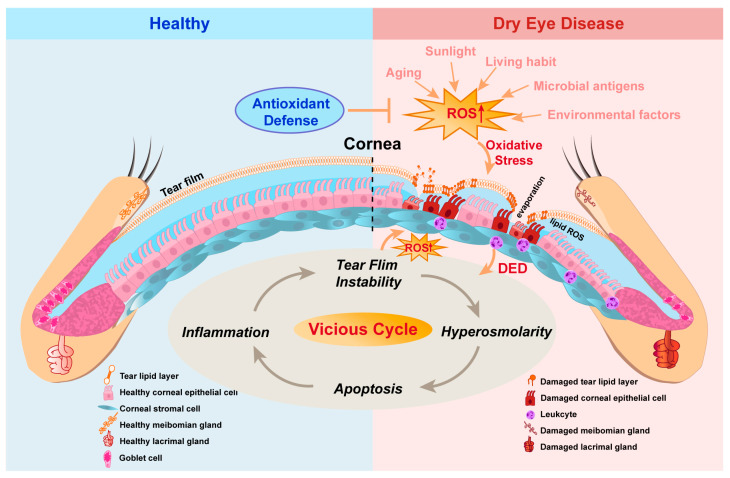
The dual roles of ROS in pathogenesis of dry eye disease (DED). The equilibrium between antioxidants and reactive oxygen species (ROS) is imperative for maintaining the health and function of ocular tissues. Several factors have the potential to elevate ROS accumulation in the tear and ocular surface, leading to oxidative stress and facilitating the progression of DED. This, in turn, exacerbates the disease as the vicious cycle of dry eye continues to generate more ROS, further deteriorating the condition.

DED is a prevalent ocular surface disorder characterized by loss of tear film homeostasis that leads a self-perpetuating cycle of ocular surface inflammation and damage [1,10]. Oxidative stress is regarded as playing a pivotal role in the pathogenesis of DED and results from an imbalance between ROS production and antioxidant capacity (Figure 1). Because of its external location, the eye is especially exposed to several external factors that may induce excessive oxidative stress on the ocular surface, such as pollutants, ultraviolet (UV) radiation, and ozone [33,34]. In addition, DED is strongly associated with aging and lifestyle factors, as excess ROS accumulates with older age, high-fat diet, and sleep deficiency [4,35,36]. Consequently, ROS accumulation can instigate DNA damage, lipid peroxide buildup, and protein modification, ultimately resulting in cell death and the infiltration of inflammatory cells into the ocular surface [37,38].

As described in earlier studies, there is increased expression of lipid peroxidase, myeloperoxidase, and xanthine oxidoreductase/xanthine oxidase in the ocular surface of DED patients. A concurrent reduction in the expression of antioxidant enzymes in DED significantly contributes to the progression of oxidative damage to the anterior ocular surface [39,40]. Subsequently, elevated levels of the main lipid hydroperoxide biomarkers have been detected in both the tear film and ocular surface of DED patients [41]. Furthermore, the increased concentration of lipid hydroperoxides is positively correlated with various parameters, including tear film break-up time, Schirmer scores, vital staining scores, conjunctival goblet cell density, and symptom scores [41]. Meanwhile, molecular indicators of oxidative stress have also been observed in animal and in vitro DED models [33]. 

Undern normal conditions, various antioxidants present in tear film can protect the ocular surface against certain radicals, such as arising from ascorbic acid, lactoferrin, uric acid, and cysteine [42]. The tear film is composed of a lipid layer, an aqueous layer, and a mucous layer, which cover the ocular surface [43]. Positioned as the outermost surface, the lipid layer is primarily produced by meibomian glands, functioning to mitigate tear evaporation [44]. The lacrimal gland produces the bulk of the aqueous layer, and the mucins in the mucous layer are mainly derived from conjunctiva and cornea. The disruption of tear film homeostasis arises when there is dysfunction of one or more of the ocular structures responsible for creating and regulating tear film components [1,3]. The close association between oxidative stress and dysfunction in both the lacrimal and meibomian glands has been demonstrated in studies. Insufficient antioxidant capacity may initiate inflammation, leading to structural impairments in both the meibomian and lacrimal glands [28,45,46]. Therefore, with the disruption of tear film homeostasis and the weakening of antioxidant defenses, the ocular surface is no longer protected.

The activation of mitogen-activated protein kinases (MAPKs) and nuclear factor-*k*B (NF-*k*B), which follow ROS-induced lipid peroxidation and occur in response to cellular stresses, leads to the production of matrix metalloproteinases (MMPs) and cytokines such as tumor necrosis factor (TNF-α), interleukin (IL)-1α, IL-1β, and MMP-9 [16,47]. In ocular tissues, this inflammatory setting induces the maturation of antigen-presenting cells (APCs) with the release of additional proinflammatory cytokines, causing epithelial cell damage [48]. Furthermore, activated APCs induce an adaptive immune response, rendering inflammation in DED more susceptible to exacerbation [11]. Notably, proinflammatory cytokines such as TNF-α possess the capacity to produce ROS, thereby intensifying oxidative stress and perpetuating the vicious cycle of ROS accumulation [49]. Taking these data together, it becomes evident that ROSs play the dual roles of both instigators and perpetuators, emphasizing the importance of their targeted inhibition as a vital element in strategies for DED treatment.

## 3. Oxidative Stress Biomarkers in DED

**Figure 2:**
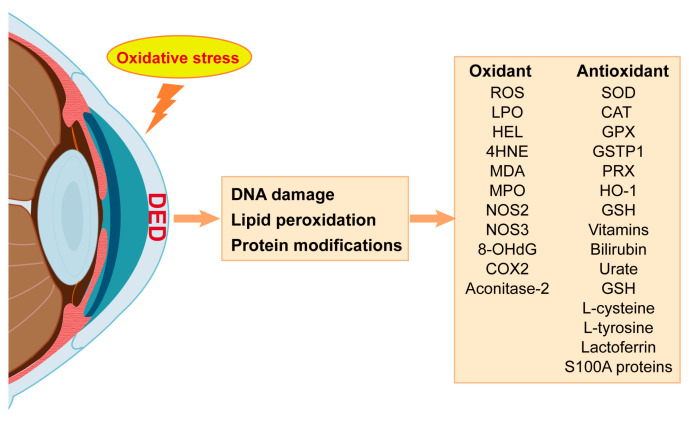
Summary of oxidative stress markers in DED.

Oxidative stress biomarkers are biological molecules or indicators employed to evaluate the degree of oxidative stress. Evidence from DED laboratory models and individuals has highlighted an imbalance in redox homeostasis as a crucial pathological factor in DED [33]. The characterization of changes in oxidative stress biomarkers can provide a reference for evaluating the progression of DED (Figure 2).

ROS, an important byproduct of mitochondrial oxidative metabolism, is a potent indicator of oxidative stress. One meta-analysis showed that markers of oxidative stress, including ROS, are elevated in the tears and conjunctiva of DED patients [50]. Moreover, air pollution has been reported to trigger ROS production in the conjunctiva and cornea, contributing to the development of DED [51]. The unique physiological conditions of the ocular surface contribute to the production of ROS and oxidative stress. Accumulation of ROS free radicals leads to lipid peroxidation of membranes, oxidative protein modification, and oxidative DNA damage [52]. Oxidant damage initiates subsequent inflammation and cell cycle disturbances, resulting in ocular surface dysfunction [53].

Cell membranes and organelles are mainly composed of lipids, which are sensitive to oxidative stress. The primary consequence of ROS overproduction is lipid peroxidation, resulting in toxic endogenous aldehydes and their derivates such as lipid peroxide (LPO), hexanoyl-lysine (HEL), 4-hydroxy-2-nonenal (4-HNE), and malondialdehyde (MDA) [29,38]. Wakamatsu, T.H. reported higher HEL and 4-HNE levels in Sjögren-related DED patients [38], and Choi W reported elevated HEL, 4-HNE, and MDA in non-Sjögren-related DED [41]. Additionally, some oxidant enzymes have been found to be overexpressed in DED, such as myeloperoxidase (MPO), nitric oxide synthase (NOS2, NOS3), and xanthine oxidase/oxidoreductase [50].

As the main aerobic metabolic sites of cells, mitochondria provide energy and are susceptible to oxidative stress. It is well known that damage to mitochondria is associated with numerous pathological processes that affect cell survival, such as apoptosis, autophagy and plasticity [54]. Oxidative DNA damage is usually assessed based on 8-hydroxy-2′-deoxyguanosine (8-OHdG), one of the predominant forms of free radical-induced oxidative lesions in nuclear and mitochondrial DNA. ROS free radicals directly induce 8-hydroxylation-specific enzymatic cleavage of guanine bases in mitochondrial and nuclear DNA [55,56]. Aconitase-2 is essentially a multifunctional enzyme involved in the tricarboxylic acid cycle [57] that is overactivated when mitochondrial activity increases under oxidative stress [58,59]. It could therefore be used as a biosensor for the redox regulation of metabolism by O_2_ to protect mitochondrial DNA from oxidative damage. The induction of increased expression of both aconitine-2 and 8-OHdG has been observed in corneal epithelial cells in response to hyperosmolarity [23]. These biomarkers are widely used as reliable indicators of oxidative damage in DED. Moreover, excessive free radical production disrupts redox status, potentially modulating the expression of various immune and inflammatory molecules, thereby instigating inflammatory processes and influencing tissue damage [60]. Cyclooxygenase-2 (COX2) is an inducible enzyme that is stimulated by lipid peroxidation. As indicated in one report, COX2 expression increases in response to oxidative stimuli in DED, mediating oxidative DNA damage and contributing to local inflammation [61]. 

The ocular surface requires robust antioxidant defenses and expresses both non-enzymatic and enzymatic antioxidants to safeguard against ROS. Non-antioxidative enzymes include glutathione (GSH), vitamins C and E, bilirubin, urate, L-cysteine, and L-tyrosine [62]. Additionally, the ocular surface is dependent on antioxidative protection provided by proteins such as lactoferrin and S100A proteins as well as antioxidative enzymes, including superoxide dismutase (SOD), catalase (CAT), glutathione peroxidase (GPX), glutathione S-transferase P (GSTP1), and heme oxygenase-1 (HO-1) [33,60]. However, a substantial alteration in antioxidant-related biomarkers in DED is attributed to oxidation–antioxidant imbalance, with a study finding that patients with DED had lower levels of lactoferrin in tears [63]. In addition, studies have shown that the uptake of lactoferrin can reduce inflammation, prevent corneal damage, as well as promote wound healing [64,65]. A deficiency in antioxidant enzymes can result in significant oxidative stress-related ocular damage [66]. Decreased SOD in the tears of DED patients has been reported [67], and mice lacking SOD1 exhibit meibomian gland dysfunction, elevated levels of 4-HNE and 8-OHdG, and inflammatory cell infiltration in the meibomian acinar epithelium [46]. In addition, antioxidant defense enzymes exhibit adaptive accommodation in response to oxidative stress exposure. 

## 4. Pathogenic Roles of Oxidative Stress in Experimental DED Models

### 4.1. Oxidative Stress in DED Animal Model Studies

Given the intricate nature of DED as a complex, chronic disease with diverse exogenous and endogenous factors, creating an animal model that accurately simulates all its pathological characteristics presents a considerable challenge. As of today, numerous animal models for DED related to oxidative stress have been developed (Table 1). Their application markedly advances our understanding of the pathogenic influence of oxidative stress in various forms of DED.

#### 4.1.1. Transgenic Animal Models

Animal models of DED should simulate the histological features observed in human DED eyes. Researchers have developed a variety of transgenic animal models to study DED, including through targeted mutations of several key factors in redox regulation.

SOD is a crucial member of the antioxidant enzyme family expressed as three isoforms. SOD1 is widely distributed in tissues and accounts for 90% of total SOD activity [68]. In SOD1 knockout mice, oxidative stress-related damage has been observed in the lacrimal glands, meibomian glands, and conjunctiva. This damage manifests as vesicle shrinkage with fibrosis, increased inflammatory cell infiltration, accelerated oxidative lipid and DNA damage, and increased apoptosis, ultimately leading to the development of DED [46,66,69]. These changes are progressively exacerbated with age, leading to decreased tear production, which further exacerbates damage to the ocular surface epithelium [66]. The transcription factor nuclear factor erythroid2-related factor 2 (NRF2), which is highly expressed in the cornea and conjunctiva, plays a crucial role in maintaining redox homeostasis by regulating antioxidant enzymes [70,71]. After acute smoke exposure, Nrf2 knockout (*Nrf2^−/−^*) mice develop several DED-like features such as decreased tear film stability, ocular surface damage, and conjunctival phenotypic changes [70]. Thus, reduced antioxidant capacity emerges as a crucial factor in the development of DED. Furthermore, oxidative stress leads to mitochondrial oxidative damage and induces dysfunction of the mitochondrial respiratory chain. The mev-1 gene induces excessive oxidative stress and was found to be the cause of DED in a mev-1 transgenic mouse model in which the presence of excess O_2_^−^ in the lacrimal glands, mass infiltration of inflammatory cells, and lacrimal gland fibrosis led to lacrimal gland dysfunction [72]. These findings suggest that oxidative stress may be a causative factor in the development of DED. 

Beyond direct regulators of oxidative stress, other animal DED models associated with abnormal tear secretion have shown that it is also intricately linked to oxidative stress. The parasympathetic nervous system plays a key role in the regulation of tear secretion by activating muscarinic acetylcholine receptors. M_3_ receptor knockout (*M3R^−/−^*) mice were used to investigate the role of muscarinic receptor subtypes in mediating tear secretion. The findings demonstrated that *M3R^−/−^* mice exhibit diminished tear secretion and display characteristic ocular surface changes reminiscent of age-related DED, such as alterations in corneal epithelial cell texture, reduced density of conjunctival goblet cells, and elevated levels of oxidative stress [73]. 

#### 4.1.2. Non-Transgenic Animal Models with Lifestyle and Environmental Changes 

In addition to transgenic animal models, nontransgenic animal models in which DED is induced by external factors are commonly used in research to explore the role of oxidative stress in different types of DED.

Dietary habit changes are closely linked to the development of DED. In high-fat diet animal models, increased expression of the ROS-generating enzyme NADPH oxidase 4 and the ROS product 3-nitrotyrosine (3-NT) at the ocular surface in addition to increased apoptosis, which destabilizes the corneal epithelial barrier function, also induces abnormalities in lacrimal gland lipid accumulation, inflammation, lipid peroxidation, and apoptosis, resulting in decreased tear secretion, which leads to DED [35,74]. This provides new insights into the mechanism of diet-related DED and new therapeutic strategies for the treatment of aging DED patients.

The extensive use of video terminals has emerged as a prevalent factor contributing to the growing prevalence of DED [75]. Light-emitting diodes (LEDs) serve as a common light source in computers and smartphones and are characterized by the significant presence of blue wavelength light. Researchers have observed that blue light LED, in contrast to red or green light, can exacerbate clinical parameters in mice with DED, leading to a significant increase in ROS and MDA production on the ocular surface and inducing oxidative damage [76]. Sleep deprivation is another consequential outcome of the changing lifestyle habits among contemporary individuals. Using a sleep-deprived mouse model, it was discovered that ROS levels increase in the lacrimal gland while the antioxidant capacity decreases and corneal epithelial squamous metaplasia is induced, ultimately leading to DED [36,77].

Smoking is a notable global public health concern and is implicated in the development of eye disease. In one study, rats exposed to cigarette smoke exhibited damage to the corneas and lacrimal glands, probably through DNA oxidation by ROS [78]. This highlights the deleterious impact of cigarette smoking on ocular surface oxidative stress and damage. Moreover, urban particulate matter, identified as another environmental factor, has been recognized as a contributor to oxidative stress on the ocular surface, leading to decreased tear production, elevated corneal irregularity, and the development of DED [79]. 

#### 4.1.3. Non-Transgenic Animal Models with Drug Induction

Drug-induced animal models are commonly utilized in DED research, in which the relationship between oxidative stress and DED is highlighted. Scopolamine, a cholinergic drug, modulates neurotransmission and suppresses tear secretion. In adult mice, DED may be induced via subcutaneous injection of scopolamine hydrobromide and exposure to low airflow and 30% humidity [80]. These animal models often simulate early mild to moderate DED, where an increased number of conjunctival 4-HNE-positive cells and heightened ROS production were observed. Patients with DED often topically apply an agent to supplement or stabilize tear film. However, BAC, a preservative commonly found in these agents, has the potential to induce damage to the ocular surface. Studies involving the administration of BAC eye drops in mice, rats, and rabbits have demonstrated its ability to induce DED [81,82]. In rats, the administration of BAC resulted in dry eye symptoms, reduced tear production, and shorter tear film break-up time in addition to decreased levels of GPX, lactoferrin, and nitric oxide (NO), exhibiting significant impacts on oxidative stress markers [82]. Furthermore, DED appears to be a significant ocular complication associated with general anesthesia based on clinical observations [83]. In a rabbit model exposed to extended periods of general anesthesia-induced DED, there was a notable decrease in tear secretion coupled with changes in tear antioxidants [84]. 

#### 4.1.4. Non-Transgenic Animal Models with Surgical Intervention 

The pathophysiological characteristics of DED can be simulated using specifically targeted surgical intervention, and an association between oxidative stress and DED was also identified in animal models subjected to surgical intervention. The lacrimal gland is the main source of tear fluid. Unilateral lacrimal gland excision induces DED in rats, leading to reduced tear secretion and corneal erosion. Simultaneously, removal of the lacrimal gland leads to elevated oxidative stress, demonstrated by increased levels of corneal 8-OHdG and HEL during the postoperative period [85]. Hormonal changes significantly contribute to maintaining the stability of the ocular surface. The female sex is regarded as a risk factor for DED, particularly in the postmenopausal stage. Reduced estrogen levels during menopause contribute to the development of evaporative DED [86,87]. In rats, oophorectomy suppresses hormone levels, and the absence of hormones induces oxidative stress, leading to decreased tear production and compromised antioxidant defenses, ultimately resulting in DED [88].

#### 4.1.5. Aging and Metabolic Disease Animal Models 

Aging is a major risk factor for many eye diseases. As individuals age, they may experience increased susceptibility to DED due to alterations in the ocular surface, leading to a decrease in both tear production and lacrimal gland function [89]. In an aging group of rats, the lacrimal gland shows increased accumulation of lipofuscin-like substances, indicating age-related oxidative damage, while the levels of vitamin E, which has antioxidant capacity, are lower. Furthermore, there is a decrease in the expression of Rab3d and Rab27b proteins involved in vesicular transport and cytosolic uptake [90]. Aging alters the expression of cytokinesis-related proteins and oxidative stress biomarkers in the lacrimal gland, which controls the expression of its downstream signaling partners, affects tear secretion, and increases susceptibility to DED.

Findings from animal studies involving induction of diabetes have also provided additional evidence supporting the association between oxidative stress and DED. In diabetic mice, oxidative stress is promoted by downregulated expression of the stress inhibitors SIRT1, FOXO3, and MnSOD. The induction of DED in db/db diabetic mice in a low-humidity chamber, accomplished by injecting scopolamine solution, results in significantly decreased tear secretion, increased corneal fluorescein staining score, and significantly increased corneal oxidative stress [91]. Similarly, in the lacrimal glands of mice where diabetes is induced by intraperitoneal injection of streptozotocin, there is a reduction in the expression levels of CAT, GPX3, and HO-1. Concurrently, the levels of oxidative stress are increased, indicating a clear link between DED and oxidative changes [92]. 

In vivo models closely resemble the physiological state of humans, including the complex cellular interactions, tissue structures, and biochemical reactions. However, they possess certain limitations. The establishment and maintenance of transgenic animal models demands considerable time and expense, while systemic gene knockout may affect other biological systems. Externally induced animal models often exhibit notable individual differences. When using drugs to construct such models, precise consideration of timing and dosage is imperative to prevent detrimental and irreversible effects. Models of animals where surgical induction is used typically entail direct trauma, resulting in their higher mortality rate. Finally, it is important to acknowledge that while in vivo models can closely approximate the physiological state, disparities in terms of the pathological processes persist between the animal models and humans with DED. Therefore, the efficacy of drugs in animal models may not necessarily be correlated with the therapeutic effects in humans with DED. Additionally, ethical considerations arise concerning the use of animal models, necessitating rigorous ethical review and adherence to moral and legal standards.

**Table 1:** Oxidative stress in DED animal models.

Model Classification	Animal Used	Index of Oxidative Stress	Changes in DED	Reference
Transgenic Animal Model	*Sod1^−/−^* mice	8-OhdG, and 4-HNE	Increase	[46,66,69]
	*Mev-1^−/−^* mice	8-OHdG	Increase	[72]
	*Nrf2^−/−^* mice	8-OhdG	Increase	[70]
	*M3R^−/−^* mice	ROS and NADPH oxidase (NOX1, NOX2, NOX4)	Increase	[73]
Non-transgenic animal models with lifestyle and environmental changes	High-fat diet mice	NOX4, MMP3, MMP9, and 3-NT	Increase	[35,74]
	Caloric restriction rats	8-OhdG, and 4-HNE	Decrease	[93]
	Blue light-exposed mice	ROS and MDA	Increase	[76]
	Sleep deprivation mice	H_2_O_2_, GPX, GSH and LPO	H_2_O_2_ and LPO increase; GPX and GSH decrease	[36,77]
	Mainstream cigarette smoke rat	ROS and 8-OhdG	Increase	[78]
	Rats exposed to urban particulate matter	8-OhdG and ROS	Increase	[79]
Non-transgenic animal models with drug induction	Mice with scopolamine hydrobromide injection in combination with exposure to low airflow and 30% humidity	4-HNE and ROS	Increase	[80]
	Rabbits administered prolonged general anaesthesia	GPX and SOD	GPX increase; SOD decrease	[84]
	Drops of benzalkonium chloride in mice, rats and rabbits	GPX, LTF and NO	Decrease	[82]
Non-transgenic animal models with surgical intervention	Unilateral excision of the lacrimal gland in rats	8-OHdG and HEL	Increase	[85]
	Postmenopausal rat	MDA, total SOD and GPX	MDA increase. Total SOD was no significant changes; GPX increase	[94]
Aging and metabolic disease animal models	Aging rat	MDA and vitamin E	MDA with significant changes; vitamin E decrease	[90]
	db/db mice injected with scopolamine solution combined with low humidity	SIRT1, FOXO3, and MnSOD	Decrease	[91]
	Mice injected with streptozotocin and placed in a smart controlled environment system with a fan	CAT, GPX3 and HO-1	Decrease	[92]

### 4.2. Oxidative Stress in DED In Vitro Model Studies

As understanding of the role of oxidative stress in the pathogenesis of dry eye deepens; numerous in vitro models have also been established in addition to animal models. Corneal and conjunctival epithelial cells are commonly used in investigating the correlation between oxidative stress and DED. Additionally, 3D corneal tissue and meibomian gland tissue cultures serve as supplementary tools to investigate the role of oxidative stress in DED (Table 2). 

Tear hyperosmolarity is a crucial step in the pathogenesis of human DED [1]. The hyperosmotic culture model of human corneal epithelial cells (HCECs) is widely acknowledged as a common in vitro model for investigating DED. In the hypertonic model of HCECs, hyperosmolarity induces oxidative damage to the ocular surface epithelium by stimulating ROS generation and increasing the expression and production of HMOX1 and COX2 enzymes [23]. Further studies in the established hypertonic model of HCECs have revealed that oxidative stress induces the increase in age-related markers and initiates mitochondrial mitophagy [95,96]. Furthermore, preservatives are used in ocular medications to prevent microbial contamination, with BAC being the most frequently used preservative in ophthalmic drugs. Additional studies have explored the impact of BAC on conjunctival epithelial cells, revealing that BAC potentiates cytotoxic and oxidative stress in conjunctival epithelial cells, particularly in conditions of hyperosmolarity. Hyperosmolarity and BAK induce the release of different proinflammatory mediators and, in combination, result in the release of additional inflammatory cytokines [24,97]. The in vitro hyperosmolarity model emphasizes the importance of avoiding BAC in DED patients.

Diabetes is one of the leading causes for the development of DED, with several studies highlighting the connection between diabetes mellitus and DED [98,99,100]. When present in culture media, high concentrations of glucose can induce oxidative stress in cultured rabbit corneal epithelial cells (RCECs) [101]. In that study, RCECs exposed to high glucose levels demonstrated increased ROS production compared with the normal group. Moreover, the generation of ROS led to oxidative stress and endoplasmic reticulum (ER) stress in RCECs, ultimately culminating in cellular apoptosis [101]. These findings offer a theoretical foundation for further investigations into diabetes-related DED.

There is clinical evidence indicating that the onset of signs and symptoms associated with DED is correlated with alcohol consumption [102]. Exposing HCECs to clinically relevant doses of ethanol results in a dose-dependent increase in cellular oxidative stress [103], accompanied by significantly increased NFE2L2 expression along with downstream antioxidant gene expression, concomitant with heightened NF-*k*B signaling. However, the proliferative characteristics of HCECs hinder the testing of chronic alcohol exposure in vitro. Moreover, these manifestations may diverge from those observed in primary cells.

However, the limitation of these in vitro models is that they cannot fully replicate patient conditions. This is because they are based on a single cell type and lack tear film, blood vessels, other cells, and air flow, all of which interact though complex multifactorial mechanisms. Thus, the development of in vitro culture system for a multilayered 3D tissue construct emerges as a viable solution to this challenge of simulating an environment closely mirroring in vivo conditions [104]. Within a 3D tissue culture system, various types of oxidative damage models can be constructed using a model of 3D-HCE tissues [105]. The 3D-HCE tissues were exposed to desiccating stress conditions (DSCs) to reproduce the corneal epithelial effects of DED in vitro. By adjusting the temperature, relative humidity, and incubation time at DSC, it is possible to modulate DED severity and the response of the corneal epithelium when exposed to different oxidative stress conditions. This culture system can be used to further substantiate the pivotal role of oxidative stress in the progression of DED, and it has the advantage of facilitating the investigation of various oxidative stress factors and their impacts on the ocular surface. Additionally, the model is amenable to high-throughput screening for drug candidates, which is helpful for identifying novel compounds and drugs to treat ocular diseases and thereby circumvent the excessive use of animals.

Oxidative stress has the potential to induce damage to meibomian gland tissue, contributing to the development of meibomian gland dysfunction (MGD) [106]. In a recent study using a Transwell chamber-assisted method, it was demonstrated that tissue viability and morphology were enhanced when rat meibomian gland explants were cultured under air–liquid interface (airlift) conditions compared with submerged conditions [107]. The levels of oxidative stress notably increased during the culturing process, showing significantly higher levels of ROS and 4-HNE in explants cultured for 24 and 48 h compared to fresh tissue. These observed changes could potentially exacerbate the pathological progression of MGD and contribute to the development of DED. This model represents a valuable tool for studying the relationship between MGD and oxidative stress. Since the functions of MG in vivo are affected by various factors, including hormones, sex, and growth factors, among others, it is necessary to optimize the culture conditions to extend survival time and enhance physiological status in the future.

**Table 2:** Oxidative stress in DED in vitro models.

In Vitro Models	Culture Condition	Oxidative Stress Features	Reference
HCECs	Hyperosmolarity	Increasing ROS production; Upregulation of age-related markers; Mitochondrial fission and mitophagy.	[23,95,96]
Conjunctival epithelial cell line	Low concentrations of BAC	Increasing superoxide anion and ROS production, induction of cell death.	[24,97]
RCECs	High-concentration glucose	Increasing ROS production, induction of cell death.	[101]
HCECs	Clinically relevant doses of ethanol	Induction of cellular oxidative stress and upregulation of antioxidant enzymes.	[103]
Human cornea	3D culture system	Epithelial tissue dehydration and cornification.	[104,105]
Rat MG explants	A transwell chamber-assisted method under airlift conditions	Increasing ROS production, induction of lipid oxidative stress.	[107]

## 5. Oxidative Stress and DED in Clinical Studies

The intricate molecular mechanism underlying oxidative stress and its association with DED has been revealed through a comprehensive examination of DED experimental models. Furthermore, clinical studies have extensively investigated the connection between oxidative stress and the severity as well as pathological manifestations of DED in affected patients (Table 3).

Over the past two decades, tear and conjunctival impression cytology samples have become a source of biomarkers in DED studies due to the minimally invasive and simple sample collection procedures [108]. In clinical practice, the oxidative stress index (OSI) is commonly calculated by quantifying the total antioxidant status (TAS) and total oxidative status (TOS) based on the levels of factors in tears. Alternatively, the degree of DED severity and efficacy of drugs are evaluated by detecting specific markers of oxidative stress [67,109]. The expression levels of 4-HNE and MDA, markers of the late lipid peroxidation in the tear or conjunctiva, are found to be associated to multiple ocular status scores, including tear film break-up time, Schirmer test, and goblet cell density, which can reflect the severity of non-Sjögren’s syndrome DED [41]. Moreover, DED is an age-related disease, with symptoms more pronounced in the elderly. The expression of MDA, a marker of oxidative stress, has been reported to be higher in older individuals. Additionally, the level of ROS in the conjunctival epithelium was found to reflect increased production of cellular ROS at the ocular surface after prolonged smartphone use. Therefore, changes in oxidative stress-related proteins may reflect an underlying mechanism for the aggravation of DED. Furthermore, the levels of LPO, SOD, CAT, and GPx as markers of oxidative stress have been reportedly used to evaluate the efficacy of eye drop treatments [67,110,111]. Such analyses have been improved due to technological advances in proteomics, and the findings have immensely contributed to the detailed understanding of the tear proteome in DED [109]. The untargeted proteomic technique facilitates the diagnosis, prognosis, and monitoring of DED [112]. Techniques can be used together to distinguish DED features based on their association with other complications such as SS, diabetes, and MGD [113]. The proteomic patterns have been studied in two groups, a DED group and an SS with DED group using 2D LC–nano-MS/MS-based proteomics, where those proteins particularly involved in stress responses exhibited increased levels in both groups. With further analysis, the levels of S100A8 and S100A9 were found to be upregulated in Sjögren’s syndrome patients with DED, whereas the levels of lactoferrin and peroxiredoxin 2 (Prx2) decreased [22]. Human conjunctival impression cytology samples of ocular surface diseases, including MGD, DED, and healthy controls, were subjected to proteomic analysis using 2D-DIGE. The discriminative protein spots were identified using MALDI-TOF/TOF mass spectrometry, with the finding that alterations in the MGD proteome are associated with oxidative stress and anti-apoptotic processes [106]. Additionally, LC–MS/MS with iTRAQ labeling was used for quantitative analysis of tear fluid samples from the three groups, with the observation that LPO, associated with oxidative stress responses, was significantly overexpressed in the MGD group [114]

Consequently, it can be concluded that oxidative stress plays an active role in the clinical progression of DED. The imbalance between oxidants and antioxidants leads to ROS release and oxidative stress. The accumulation of ROS triggers inflammation, subsequently leading to ocular surface damage [37,38]. It is widely accepted that the progression of DED is due to a vicious circle of inflammation, whereby oxidative stress exacerbates this cycle [3]. The connection between oxidative stress and inflammation in DED has also been further verified in clinical studies.

In conjunctival samples of individuals with SS, increased numbers of HEL and 4-HNE positive cells as well as inflammatory cell density were observed. Additionally, there was also an increase in HEL levels in tear samples [38]. This indicates that ROS production, lipid peroxidation-related membrane damage, and inflammation are closely interrelated. Further clinical investigation confirmed that ROS can activate NLRP3 inflammasomes to induce inflammation in individuals with DED via the ROS–NLRP3–IL-1β signaling pathway [115]. This serves as additional evidence that oxidative stress promotes the progression of inflammation.

In recent years, the significance of oxidative stress is being increasingly recognized by clinicians, as it stable and can be easily and efficiently detected. Moreover, tear film and conjunctival impression cytology samples are relatively convenient to harvest from DED patients, which makes it easier to detect oxidative stress markers. Changes in the level of markers can reflect disease progression, with the potential for markers to be used for clinical DED staging in the future, enhancing the appropriate selection of relevant clinical treatment. Meanwhile, the changes in oxidative stress markers can used as indexes for the evaluation of drug efficacy, which serve as effective reference standards for clinical treatment. Clinical studies have expanded the application prospects of oxidative stress in DED, which may contribute to laying a foundation for promoting mechanism research, improving diagnosis, and the development of therapeutic drugs in the future.

**Table 3:** Oxidative stress in DED patients.

Description	Oxidative Stress Clinical Parameters	Changes in DED	Reference
Tear film	The level of lactoferrin	Decreased	[22]
	The level of S100A8 and S100A9.	Increased	[22]
	The level of Peroxiredoxin 2 (Prx2)	Decreased	[22]
	The expression of SOD, CAT and GSH-Px	Decreased	[67]
Conjunctival impression cytology samples	The level of ROS	Increased	[29,115]
	The level of LPO	Increased	[110]
Conjunctival impression cytology samples/tear film	The numbers of HEL and 4HNE positive cells of conjunctiva; Tear concentrations of HEL	Increased	[38]
	The expression of 4-HNE and MDA	Increased	[41]
	The levels of LPO	Increased	[110,116]

## 6. Therapeutic Roles of Antioxidants in Oxidative Stress in DED

### 6.1. Antioxidant Therapeutic in Clinical Studies

As a crucial factor in the pathogenesis of DED, as of recent, oxidative stress is considered as an effective target for its treatment. Lactoferrin, secreted by lacrimal gland into tears, functions as an antioxidant to protect ocular cells against cell death caused by oxidative stress. Oral administration of enteric-coated lactoferrin tablets has proven effective in treating DED, with a significant improvement in symptoms [117]. This conservative treatment emphasizes the importance of antioxidant supplementation. Therefore, alleviating DED through antioxidant supplementation represents a novel therapeutic concept that requires further exploration. Several studies have highlighted the preventive and therapeutic approaches of antioxidants in patients with DED (Table 4), where some are under investigation or in early-phase clinical trials. 

SkQ1, a mitochondria-targeted antioxidant, is able to target and neutralize mitochondrial ROS. Its effectiveness and safety in protecting ocular patients with DED against corneal damage have been demonstrated [118]. As a highly potent antioxidant, N-acetylcysteine (NAC) has been shown to exert beneficial therapeutic effects on DED. A controlled, randomized, double-blind clinical investigation confirmed the clinical improvement from chitosan-N-acetylcysteine (C-NAC) treatment, evidenced by enhanced OSDI scores, decreased corneal damage incidence, and reduced frequency of symptoms related to ocular discomfort and conjunctival redness within 5 days of C-NAC treatment [119]. Beyond the direct use of antioxidants for treatment in the clinic, other treatments can also enhance antioxidant capacity and improve dry eye symptoms. Cataracts are closely associated with oxidative stress, and dietary antioxidants are currently recognized for their potential preventive effects [120]. Cataract surgery is one of the most common ophthalmic surgeries; it causes corneal nerve denervation, resulting in impaired epithelial wound healing, increased permeability, and decreased metabolic activity. DED frequently arises as complication following surgery, and medication use has been demonstrated to be correlated with its incidence [121,122]. Antioxidant strategies in preoperative or postoperative ocular surface medication can mitigate the risk of complications caused by oxidative stress. In a recent study, preoperative artificial tear treatment combined with recombinant bovine basic fibroblast growth factor demonstrated significant enhancements in ocular surface function and total antioxidant capacity. This combined approach notably decreased tear inflammatory and oxidative stress factors, ultimately alleviating dry eye symptoms postsurgery in cataract patients [123]. The findings from a randomized controlled study in Korea revealed that treatment with preservative-free and preserved sodium hyaluronate 0.1% and fluorometholone 0.1% eyedrops promotes the anti-inflammatory and antioxidant effects in the tears of cataract patients with pre-existing DED after surgery [124]. In addition, another study verified the protective effects of preservative-free eye drops containing 0.15% hyaluronic acid and vitamin B12 on oxidative stress and dry eye symptoms [110].

**Table 4:** Antioxidants in DED clinical trials.

DED Patients	Study Design	Treatment	Oxidative Stress Outcome	Dry Eye Outcome	Reference
Patients with dry eye syndrome	Randomized, double-masked study over 6 weeks; N = 240.	SkQ1	-	Increased tear film stability, reduced corneal damage and dry eye symptoms	[118]
	Randomized controlled study over 5 days; N = 38	C-NAC eye drops	-	Decreased OSDI, fluorescein stain score; increased tear film thickness; reduced corneal damage and symptoms of ocular discomfort/conjunctival redness	[119]
Cataract patients complicated with DED	Randomized controlled study over 1 week; N = 118	Recombinant bovine basic fibroblast growth factor eyedrops	Decreased MDA and lipid peroxide; improved SOD and total antioxidant capacity	Decreased clinical symptom score, OSDI, fluorescein stain score, TNF-α and IL-6; improved TBUT and Schirmer I test score	[123]
Patients with DED after cataract surgery	Randomized controlled study over 2 months; N = 80	Preservative-free sodium hyaluronate 0.1% and preservative-free fluorometholone 0.1% eyedrops	Increase concentrations of catalase and SOD2 in tear	Improved OSDI score, TBUT, Schirmer I test, corneal fluorescein staining and impression cytology finding	[124]
	Randomized parallel group over 1 month; N = 103	Preservative-free hyaluronic acid 0.15% and vitamin B12 eyedrops	Reduced lipid peroxidation (LP-CHOLOX test)	Decreased OSDI and FCT scores; increased Schirmer test and BUT scores	[110]

### 6.2. Other Antioxidants in Protecting against DED

Apart from the aforementioned antioxidant drugs currently undergoing clinical research, numerous other compounds identified for their promising antioxidant potential in basic research have demonstrated effectiveness in alleviating dry eye symptoms in experimental models by mitigating oxidative stress (Table 5).

Using in vitro DED models, various substances have been found to exhibit favorable antioxidant effects, such as 0.2% xanthan gum, which shields HCECs from oxidative stress, allowing ROS levels to be restored to normal values [125]. In H_2_O_2_-treated rabbit corneal epithelial cells, taurine significantly reduced ROS production [126]. Calcitriol [15], L-carnitine [127], and zidovudine [128] can also significantly attenuate hyperosmotic stress-induced oxidative stress and cellular inflammation in HCECs. In addition, there are numerous plant extracts that significantly attenuate oxidative stress in in vitro dry eye models, such as olive pomace extracts [129], thymoquinone [130], and pterostilbene [131].

Numerous studies have highlighted the protective effects of potential antioxidant medications in experimental DED animal models. Tear production by the lacrimal gland is influenced by neuroendocrine, hormonal, and immunological factors, and ROS plays an important role in its regulation. One study found that ovariectomized rats treated with alpha-lipoic acid (ALP) had increased lacrimal peroxidase activity and improved tear production [94]. In rat models of DED, 2-hydroxy estradiol (2-OHE2), a catechol derivative of 17β-estradiol (E2), scavenges tyrosyl radicals and possibly suppresses oxidative stress in corneal epithelial cells, thereby suppressing corneal erosion [132]. The antioxidative effects of vitamin D have also been demonstrated in other studies using dry eye models [133,134]. In *Sod1^−/−^* mice, tear function and ocular surface epithelial damage scores were significantly worse than in WT mice. However, following a 2-week treatment with 2% rebamipide, there were significant improvements in tear film break-up time, tear pro-duction, and corneal epithelial damage scores, demonstrating its antioxidant effects on DED in *Sod1^−/−^* mice [135]. Moreover, several studies have highlighted the protective potential of natural antioxidant plant extracts against DED. Extracts from plants containing resveratrol [136], quercetin [137], and capsicum annum [82] have exhibited significant antioxidant properties, showcasing their potential in mitigating DED-related oxidative stress.

**Table 5:** Summary of selected experimental studies on antioxidants in DED.

Animal Used	Method of Inducing DED	Molecule Treated	Index of Oxidative Stress	Parameter of Dry Eye Studied	Reference
Mouse	*Sod1^−/−^* mice	Rebamipide	4-HNE and 8-OHdG	Improved BUT, tear production and ocular surface epithelial damage scores; increased the expressions of mucins and the density of goblet cells; reduced inflammatory cytokines level	[135]
Mouse	Subcutaneous scopolamine injection and desiccating stress	Hyaluronic Acid and Omega-3 Essential Fatty Acids	4-HNE and HEL	Improved corneal irregularity scores and corneal fluorescein staining scores; reduced inflammatory cytokines level	[138]
Mouse	BAC topical application	Resveratrol	SIRT1, GPx, and SOD2	Increased the density of goblet cells and tear production; reduced corneal fluorescein staining scores	[136]
Mouse	db/db diabetic mice	Quercetin	SOD1 and SOD2	Increased tear volume and tear production; improved lacrimal gland morphology	[137]
Rat	Ovariectomy	ALP	Total SOD, GPx, levels of carbonyl and MDA	Increased tear production	[94]
Rat	Remove the lacrimal glands	2-OHE2	Steroidal radical scavenging activity, prostaglandin endoperoxide synthase (PGS) activity	Increased tear volumes; reduced corneal fluorescein staining scores	[132]
Rat	3 mCi/kg RAI gastric gavage	Coenzyme Q10 (CoQ10)	Total oxidant status (TOS) and total antioxidant status (TAS)	Improved lacrimal gland morphology; reduced inflammatory cytokines level	[139]
		Vitamin D (calcitriol)	TOS, TAS	Improved lacrimal gland morphology; reduced inflammatory cytokines level	[133]
Rat	Benzalkonium chloride (BAC) topical application	Vitamin D3 (LCD)	MDA, SOD, and GPx	Increased tear volume BUT tear film integrity and tear protein levels; reduced ocular surface inflammation	[134]
		Capsicum annum (CCA)	GPx, NO, lactoferrin and prostaglandin-endoperoxide synthase 2 (PTGS2)	Increased tear production BUT reduced ocular surface inflammation	[82]

## 7. Conclusions and Future Prospects

In conclusion, this review underscores the critical role of oxidative stress in the pathogenesis of DED. Oxidative stress, resulting from an imbalance between ROS production and antioxidant defenses, contributes to tear film instability, ocular surface damage, and inflammation in DED patients. Biomarkers of oxidative stress provide valuable insights into disease progression and severity, highlighting the importance of monitoring and targeting these markers in clinical practice. 

Moving forward, future research should focus on innovative therapeutic approaches that target oxidative stress pathways to effectively manage DED. This may involve the development of novel antioxidants or the repurposing of existing drugs with antioxidant properties to mitigate ROS-induced damage. Furthermore, longitudinal studies investigating the association between oxidative stress biomarkers and disease progression are warranted to better understand the natural causes of DED and identify early predictive markers for severity. Collaborative efforts between basic scientists, clinicians, and industry partners are essential for translating preclinical findings into clinically relevant interventions. Finally, various factors contribute to the development of DED, primarily including aging, hormonal changes, and lifestyle issues, which are all related to the generation of oxidative stress. Therefore, individuals should pay closer attention to the impact of these factors. Furthermore, raising awareness about the role of oxidative stress in DED among healthcare providers and patients is crucial for promoting early diagnosis and effective management strategies. By addressing oxidative stress pathways, we can pave the way for improved therapeutic outcomes and enhanced quality of life for individuals affected by DED.

## Data Availability

Not applicable.

## Abbreviations

DED	dry eye disease
ROS	reactive oxygen species
LPO	lipid peroxide
HEL	hexanoyl-lysine
4-HNE	4-hydroxy-2-nonenal
MDA	malondialdehyde
MPO	myeloperoxidase
NOS	nitric oxide synthase
8-OHdG	8-hydroxy-2′-deoxyguanosine
COX2	cyclooxygenase-2
GSH	glutathione
SOD	superoxide dismutase
CAT	catalase
GPX	glutathione peroxidase
GSTP1	glutathione s-transferase P
HO-1	heme oxygenase-1
3-NT	3-nitrotyrosine
BAC	benzalkonium chloride
NO	nitric oxide
HCECs	human corneal epithelial cells
RCECs	rabbit corneal epithelial cells
ER	endoplasmic reticulum
MGD	meibomian gland dysfunction
OSDI	ocular surface disease index
FCT	fluorescein clearance test
BUT	break-up time
NAC	N-acetylcysteine
C-NAC	chitosan-N-acetylcysteine
ALP	alpha-lipoic acid
2-OHE2	2-hydroxy estradiol
